# Role of Systemic Inflammatory Response Markers in Urothelial Carcinoma

**DOI:** 10.3389/fonc.2020.01473

**Published:** 2020-08-21

**Authors:** Hyeong Dong Yuk, Ja Hyeon Ku

**Affiliations:** ^1^Department of Urology, Seoul National University Hospital, Seoul, South Korea; ^2^College of Medicine, Seoul National University, Seoul, South Korea

**Keywords:** biomarker, C-reactive protein, neutrophil to lymphocyte ratio, systemic inflammation response, urothelial cancer

## Abstract

Urothelial carcinoma (UC) can occur in various parts of the urinary tract and occurs in different stages and grades. The disease recurs frequently and is monitored through a series of invasive tests, such as cystoscopy or ureteroscopy, over the lifetime of an individual. Although many researchers have attempted to stratify the risks of UC, with the majority being based on cancer characteristics and host factors such as performance status, a risk classification system has yet to be fully developed. Cancer affects various parts of the body through the systemic immune response, including changes in hormones, the number and ratio of white blood cells and platelets, and C-reactive protein (CRP) or albumin levels under the influence of neuroendocrine metabolism, hematopoietic function, and protein and energy metabolism, respectively. Herein, we reviewed various systemic inflammatory response markers (SIRs) related to UC, including CRP, albumin-globulin ratio, albumin, Glasgow prognostic score (GPS), modified GPS, neutrophil-lymphocyte ratio, and platelet-lymphocyte ratio. Our aim was to summarize the role of various SIRs in the treatment of patients with UC.

## Introduction

Urothelial carcinoma (UC) is the fourth most common cancer (1). The cancer is classified based on the site of occurrence as bladder UC (BC) and upper urinary tract UC (UTUC) (1–4). BC accounts for 90–95% of all UCs, (1–4) and is the fourth most common cancer among men in the United States, ranking eighth in terms of mortality (4). UC can occur in various parts of the urinary tract and in different stages and grades (2–4). Most UCs start in the bladder, and 75% of the patients are initially diagnosed with non-muscle invasive bladder cancer (NMIBC) (3, 4). Endoscopic surgery, intravesical therapy, and immunotherapy can be performed for the treatment of NMIBC (3). A combination of radical cystectomy (RC), radiation therapy (RT), chemotherapy, and immunotherapy among other treatments have been considered in the standard management of MIBC (2). UTUC demonstrates twice as many pyelocalyx as ureters. More than 60% of the patients have been reported to present with invasive cancer at the time of diagnosis and 15–25% have BC (4). Radical nephroureterectomy (RNU) is the standard treatment of UTUC. A 22–47% recurrence rate of the cancer has been reported in the bladder in contrast to 2–6% in the opposite upper tract (4). Local and distant metastases are associated with a poor prognosis despite the availability of various treatment options (2–4). A study reported that the 5-years relative survival rate of patients with BC, diagnosed from 2009 to 2015, was ~77%, with the occurrence of regional and distant metastases in 38 and 5% of the cases, respectively (1). In contrast, the upper tract variant is a relatively rare disease that accounts for 5–10% of all UCs (4). Considering the high relapse rate, the cost of treatment of BC from diagnosis to death, with repeated surgeries and investigations, is the highest among all UCs (2–4). Appropriate risk stratification and prediction of prognosis are important to determine the necessary treatment protocols based on the characteristics of UC (2–4). Accordingly, several studies have assessed the role of systemic inflammatory response markers (SIRs) in the prediction of the progression and prognosis of UC. C-reactive protein (CRP), albumin, albumin-globulin ratio (AGR), Glasgow prognostic score (GPS), modified GPS (mGPS), neutrophil-lymphocyte ratio (NLR), and platelet-lymphocyte ratio (PLR) have been studied as potential prognostic factors. Herein, we reviewed studies on different SIRs for the diagnosis and treatment of UC.

## Sir Markers in Cancer

Cancer progression and prognosis are affected by a variety of factors. Representative parameters in the treatment of cancers are the characteristics of the tumor associated with prognosis. These include the pathological variant, stratified grade and stage, and the imaging stage, which are considered in the measurement of disease progression. However, in addition to these characteristics, status of the patient, including performance status, degree of systemic inflammatory response, muscle mass, and weight status (sarcopenia or cachexia) are also important parameters to be considered. Inflammation plays an important role in the development and progression of cancers as well as in the patients' response to therapy (5). Tumors consist of various types of inflammatory and immune cells (6), which can activate immune cells to produce cytokines, chemokines, growth factors, and prostaglandins (6, 7). The inflammatory microenvironment is an essential component of tumorigenesis since most cancers trigger an inflammatory response by building a pro-tumorigenic microenvironment (6, 8). The resultant inflammation affects the host immune response to the tumor (6, 8). Potential SIR-related biomarkers in cancer patients include as CRP, NLR, PLR, albumin and GPS.

Level of CRP increases blood circulation in various immunologically mediated inflammatory conditions, including trauma, infection, surgery, burns, allergic reactions, and cancer (9). CRP is primarily synthesized in the liver and is a major component of the innate immune system. It is involved in the initial immunity during infection and in the process of cell death (10). CRP binds to phosphocholine on the surface of damaged and foreign cells and bacteria, activating phagocytosis by macrophages (10). The normal concentration of CRP in healthy adults is between 0.8 and 3.0 mg/L. However, the level of this protein increases during the acute phase of inflammation (10). Depending on the cause, levels of CRP increase to over 50–100 mg/L in 4–6 h and have also been reported to increase over 1000-fold (10). The concentration of CRP is typically maintained in the range of 2–10 mg/L in individuals with chronic inflammation (10).

It is known that secretion of CRP is primarily regulated by IL-6; however, its mechanism has not been fully elucidated. There is lack of clarity on whether elevation of CRP is related to the inflammatory factors released by the tumor or due to a compromised host immune response. Although there is no clear evidence of the diagnostic or etiological role of CRP in cancer, several studies have reported higher levels of this protein in cancer patients than in healthy individuals (11). Moreover, high baseline levels and specific single nucleotide polymorphisms of CRP have been previously associated with an increased risk of cancer (11). Many recent studies have reported an association between high CRP levels and poor prognosis as well as progressive disease in patients with a variety of cancers, including lung, kidney, colorectal, breast, and ovarian cancers (11).

NLR, another marker of reactions to inflammatory conditions. An absolute NLR ratio has not yet been defined owing to the lack of clarity of the association between high NLR and poor survival in cancer patients. In general, neutrophils provide rapid defense against microorganisms in the infected area during a superficial infection (12). Similarly, in cancers, neutrophils are displaced around tumor cells in response to cytokines and chemical attractants to participate in the initial inflammatory response (12). They recruit CD8+ T cells and promote an anti-tumor response by inducing the production of cytokines, such as TNF-α or IL-12 (12). In contrast, several phenotypes of neutrophil exist, which can promote the formation of tumors *via* their involvement in immunosuppression mediated by TGF-β (12). Neutrophils stimulated with G-CSF were found to improve the growth of circulating tumor cells in metastatic sites and were involved in angiogenesis caused by cancer (12). Many studies have been published recently on the role of NLR in patients with UC (12). A high NLR was reported to be associated with poor prognosis in cancer patients (12).

Albumin, another marker of reactions to inflammatory conditions. Serum albumin and globulin are the major components of blood plasma (13). Albumin is predominantly produced by the liver. Globulins are produced by the liver as well as the immune system. The main function of albumin is to regulate the volume of blood by maintaining the colloid pressure of blood (13). Infections, burns, liver disease, nephrotic syndrome, and malignant tumors decrease serum albumin levels (13). Normal serum albumin levels range from 3.5 to 5 g/dL and that of serum globulin are 2.6–4.6 g/dL (13). The association between albumin levels and the prognosis of patients has been reported in various cancers, including ovarian, colorectal, and lung cancers, in addition to UC (14). Globulin is composed of various pro-inflammatory proteins such as complement components and immunoglobulins and plays an important role in immunity and inflammation (14). Levels of globulin increase in patients with chronic inflammation or cancer. Albumin and globulin are associated with immune responses in cancer patients as well as the nutritional status (14). Low albumin levels reflect a decline in the status of nutrients, which is common in cancer patients, and is known to interfere with immune mechanisms such as humoral and cellular immunity and phagocytic function (14). Hypoalbuminemia in cancer patients has been reported to result from a reduction in albumin synthesis mediated by secretion of pro-inflammatory cytokines, increased catabolism, and cachexia (14). Increased vascular permeability in these patients also reduces blood albumin levels due to redistribution of the protein from the intravascular to the interstitial areas (14).

Inflammatory cytokines or chemokines are responsible for the activation of platelets (15). Activated platelets induce leukocytes to accumulate in inflamed regions (15). Platelets interact with leukocytes and induce a bidirectional cell activation process, wherein the platelets activate leukocytes, and various leukocyte-derived molecules activate platelets (15). Activated platelets stimulate neutrophils, which in turn release chromatin (15). Chromatin further recruits and activates platelets (15). Cancer cells in the bloodstream induce platelet-mediated recognition (16). Platelets are amplified by a variety of immune cells, cell products, cell surface receptors, and extracellular factors (16). Under certain circumstances, the interaction between cancer cells and platelets can suppress the recognition or removal of cancer cells by immune cells (16).

## C-Reactive Protein

Several studies on UC have reported that elevated CRP levels before surgery or chemotherapy were associated with poor prognosis (Table 1) (17–36). The cutoff value for CRP varies from 3.4 to 10 mg/L. While most studies have compared their results with the prognosis for RNU or RC preoperative evaluation (21–24, 28–34), Yoshida et al. (24), and Egger et al. (25) compared the prognosis with a pre-cancer evaluation. Yoshida et al. included 88 patients with MIBC and reported that a high level of CRP before treatment was a factor in predicting the 5-years cancer specific survival (CSS) after chemoradiotherapy (24). Egger et al. showed that CRP levels before chemotherapy in patients with metastatic BC correlated with patient- and cancer-specific characteristics. The median progression-free survival (PFS) in patients with high CRP levels was extended by 1.5 months compared to patients with low CRP levels. Furthermore, the 1-year overall survival (OS) rate was higher at 60.4% (25). Nakagawa et al. evaluated the prognosis of patients with metastatic UC based on preoperative levels of CRP (34). The study reported that higher pre-metastasectomy levels of CRP was associated with worse prognosis [hazard ratio (HR) 0.24, *P* = 0.005]. Therefore, the authors suggested evaluation of CRP levels when considering metastasectomy (34).

**Table 1:** The studies evaluating the role of C-reactive protein in urothelial cancer.

**Study**	**Year**	**Case**	**Site**	**Stage**	**Assessment and Treatment**	**Threshold**	**Outcomes**
Ishioka et al. (17)	2012	232	Bladder + Ureter	pT4 or N+ or M+	At diagnosis	5.15 mg/L	OS, HR 1.68 (1.27–2.30), *P* < 0.001 Median OS, 16.0 m (CRP ≤ 5), 6.5 m(5 < CRP ≤ 10), 3.8 m (10 ≤ CRP < 30), 2.6 m (30 ≤ CRP)
Hilmy et al. (18)	2005	105	Bladder	pTa-2	Pre-TURBT	10.0 mg/L	CSS, HR 3.31 (1.09–10.09), *P* = 0.035 OS, HR 2.73 (1.23–6.07), *P* = 0.014
Mbeutcha et al. (19)	2016	1117	Bladder	pT1N0M0	Pre-TURBT	5 mg/L	PFS, HR 1.72 (1.05–2.81), *P* = 0.031
Gakis et al. (20)	2011	246	Bladder	pT2-4N0/+	Pre-RC	5 mg/L	CSS, HR 1.18 (1.09–1.27), *P* = 0.001 3 yr CSS 44 vs. 74%, *P* < 0.0014 (high vs. low)
Kramer et al. (21)	2013	194	Bladder	pT3-4	Pre-RC	5 mg/L	CSS, HR 1.68 (1.17–2.09)
Nakagawa et al. (22)	2013	114	Bladder	pT0-4N1-3	Pre-RC	5 mg/L	OS, HR 2.63 (1.58–4.39), *P* < 0.001
Sejima et al. (23)	2014	249	Bladder	pT0-4N0/+M0	Pre-RC	5 mg/L	DSS, HR 1.99 (1.13–3.52), *P* = 0.016
Yoshida et al. (24)	2008	88	Bladder	cT2-4N0M0	Pre-Radiochemotherapy	5 mg/L	CSS, HR 1.80 (1.01–2.97), *P* = 0.046
Eggers et al. (25)	2013	34	Bladder	M+	Pre-Chemotherapy	80 mg/L	OS, HR 14.8 (3.7–60.0), *P* < 0.001 1 yr-OS: 22.2 vs. 82.6%, *P* < 0.001 (high vs. low)
Nakagawa et al. (26)	2017	1087	Bladder	pT0-4N0-3M0	Pre-Treatment	5 mg/L	OS, HR 1.48 (1.00–2.19), *P* = 0.049
Saito et al. (27)	2007	130	Ureter	pTa-4N0M0	Pre-RNU and Partial ureterectomy	5 mg/L	RFS, HR 1.45(1.05–1.97), *P* = 0.023 DSS, HR 1.78 (1.21–2.68), *P* = 0.004
Obata et al. (28)	2013	183	Ureter	pTa-4N0M0	Pre-RNU	5 mg/L	RFS, 2.83 (1.41–5.68), *P* < 0.001 CSS, 2.65 (1.24–5.65), *P* < 0.012
Stein et al. (29)	2014	115	Ureter	pTa-4N0/+M0/+	Pre-RNU	5 mg/L	5 yr CSS 26.4 vs. 54.2%, *P* = 0.006 (high vs. low) CSS, HR 2.67 (1.28–5.54), *P* = 0.009
Aziz et al. (30)	2014	265	Ureter	pTa-4N0/+M0	Pre-RNU	9 ml/L	RFS 1.18 (0.71–1.97), *P* = 0.050 DSS 1.61 (0.95–2.73), *P* = 0.026
Tanaka et al. (31)	2014	564	Ureter	pTa-4N0/+M0	Pre-RNU	≤ 5 mg/L/5.1–20 mg/L/>20 mg/L	CSS, HR 1.74 (1.15–2.64), *P* = 0.009, (5.1–20 vs. ≤ 5); HR 2.31 (1.41–3.82), *P* = 0.001, (>20 vs. ≤ 5) RFS, HR 1.47 (1.01–2.13), *P* = 0.007, (>20 vs. ≤ 5)
Morizane et al. (32)	2015	345	Ureter	pTa-4N0/+M0	Pre-RNU	5 mg/L	CSS, HR 2.43 (1.30–4.54), *P* = 0.006
Fujita et al. (33)	2015	45	Ureter	pTa-4N1-3M0	Pre-RNU / ACT	2.8 mg/L	CSS, HR 3.20 (1.21–8.43), *P* = 0.018
Nakagawa et al. (34)	2017	37	Bladder + Ureter	pT0-4N0/+M1 with metasectomy	Pre-Metasectomy	5 mg/L	CSS, HR 0.24 (0.09–0.64), *P* = 0.005 (low)
Saito et al. (35)	2012	80	Bladder + Ureter	M+	Pre-2nd line chemotherapy	Kinetics	OS, HR 2.21 (1.41–3.28), *P* = 0.001 (non-normalized)
Matsumoto et al. (36)	2018	114	Bladder + Ureter+Urethra/ Prostate	M+	Pre-2nd line chemotherapy	10 mg/L	OS, HR 2.63 (1.43–4.76), *P* = 0.002

*ACT, adjuvant chemotherapy; CRP, C-reactive protein; CSS, cancer specific survival; DSS, disease specific survival; HR, hazard ratio; OM, overall morbidity; OS, overall survival; RC, radical cystectomy; RFS, recurrence free survival; RNU, radical nephroureterectomy; TURBT, transurethral resection of bladder tumor*.

CRP is considered a clinically significant biomarker based on current evidence and a static variable in predicting prognosis. However, CRP kinetics has been considered a variable to measure prognosis; therefore, its potential as a dynamic variable has also been reported. Saito et al. measured CRP levels before and after second-line chemotherapy and not before surgery (35). The prognosis was evaluated based on the kinetics of CRP. In the umbilical evaluation, patients were divided into groups based on CRP levels greater or lesser than 5 mg/L. Patients with preoperative CRP levels of ≥5 mg/L were categorized as the non-normalized group, and those with levels ≤5 mg/L were considered the normalized group. The study reported that patients in the non-normalized group demonstrated poor prognosis before and after surgery (HR 2.21, *P* = 0.001). Tanaka et al. also compared the prognosis of individuals with CRP levels ≤ 5, 5.1–20, and ≥20 mg/L. Patients with CRP levels >20 mg/L had poor CSS and recurrence-free survival (RFS) compared to those with values ≤ 5 mg/L (HR 1.74, 1.47, *P* = 0.0009 and 0.007, respectively) (31).

## Neutrophil-to-Lymphocyte Ratio

Several papers have reported on the prognostic role of NLR in NMIBC (Table 2) (19, 37–46). Most of these studies have reported on the relationship between NLR, RFS, and PFS before transurethral resection of bladder tumor (TURBT) (19, 37–45). Vartolomei et al. and Mbeutcha et al. analyzed NLR in more than 1,000 patients (19, 43). Mbeutcha et al. reported that a high NLR was associated with RFS (HR 1.27, *P* = 0.013), which was reported to be related to PFS (HR 1.72, *P* = 0.007) (19). Similarly, Vartolomei et al. demonstrated high NLR and low NLR (58.8 and 9.4%) for 5-years RFS (*P* < 0.001), 79.2 and 57.1%) for 5-years PFS (*P* < 0.001), and 84.3 and 77.4% for 10-years CSS (*P* = 0.004) (43). A high NLR was associated with RFS (HR 3.34, *P* < 0.001), PFS (HR 2.18, *P* < 0.001), and CSS (HR 1.65, *P* = 0.030) (68). In addition, Yuk et al. reported on the relationship between OS (HR 2.24, *P* = 0.005) and CSS (HR 2.03. *P* = 0.039) based on the NLR in patients before BCG treatment (46).

**Table 2:** The studies evaluating the role of neutrophil to lymphocyte in urothelial cancer.

**Study**	**Year**	**Cases**	**Stage**	**Grade**	**Assessment and treatment**	**Threshold**	**Outcomes**
**Non-muscle invasive bladder cancer**							
Mano et al. (37)	2015	107	pTa-1	G1-3	Pre-TURBT	2.41	3 yr-PFS, 84 vs. 61%, *P* = 0.004 PFS, HR 3.52 (1.33–9.33), *P* = 0.012
Favilla et al. (38)	2016	178	pTa-1	LG/HG	Pre-TURBT	3	RFS, HR 2.84 (1.5–5.75), *P* < 0.010 PFS, HR 5.35 (0.39–73.7), *P* < 0.210
Mbeutcha et al. (19)	2016	1117	pTa-1	G1-3	Pre-TURBT	2.5	RFS, HR 1.27 (1.05–1.53), *P* = 0.013 PFS, HR 1.72 (1.16–2.54), *P* = 0.007
Ogihara et al. (39)	2016	605	pTa-1	LG/HG	Pre-TURBT	2.2	5 yr-RFS, 66.3 vs. 31.7%, *P* < 0.001 5 yr-PFS, 97.5 vs. 90.4%, *P* < 0.001 RFS, HR 2.08 (1.6–2.7), *P* < 0.001 PFS, HR 2.37 (1.17–4.78), *P* = 0.016
Ozyalvacli et al. (40)	2016	166	pT1	HG 1	Pre-TURBT	2.43	RFS, HR 3.81 (1.5–9.67), *P* = 0.005
D'Andrea et al. (41)	2017	918	pTa-1	G1-3	Pre-TURBT	3	5 yr-RFS, 55.5 vs. 45.9%, *P* < 0.010 5 yr-PFS, 94.9 vs. 89.9%, *P* = 0.004 RFS, HR 1.3 (1.1–1.6), *P* = 0.004 PFS, HR 1.9 (1.2–3.0), *P* = 0.006
Kang et al. (42)	2017	1698	pTis-1	PUNLMP/LH-HG	Pre-TURBT	2.0	OS, HR 1.52 (1.19–1.95), *P* = 0.001 CSS, HR 1.12 (1.01–1.25). *P* = 0.030
Vartolomei et al. (43)	2018	1046	pT1	HG/G3	Pre-TURBT		5 yr-RFS 58.8 vs. 9.4%, *P* < 0.001 5 yr-PFS 79.2 vs. 57.1%, *P* < 0.001 10 yr-OS 66.5 vs. 63.6%, *P* = 0.030 10 yr-CSS 84.3 vs. 77.4%, *P* = 0.004 RFS, HR 3.34 (2.82–3.95), *P* < 0.001 PFS, HR 2.18 (1.71–2.78), *P* < 0.001 CSS, HR 1.65 (1.02–2.66), *P* = 0.030
Getzler et al. (44)	2018	113	pTa-1	G1-3	Pre-TURBT	2.5	RFS, HR 2.10 (1.17–3.75). *P* = 0.012 RFS HR 3.96 (1.19–13.16). *P* = 0.025, (BCG subgroup)
Racioppi et al. (45)	2019	100	high-risk NMIBC, pTa-1	HG	Pre-TURBT	3	Recurrence risk score (*r* = 0.55, *p* = 0.01) Progression risk score (*r* = 0.49, *p* = 0.01) BCG response, OR0.08 (0.008–0.147), *P* = 0.02
Yuk et al. (46)	2019	385	pTis-1	LG/HG	Pre-BCG treatment	1.5	OS, HR 2.24 (1.26–3.96), *P* = 0.005 CSS, HR 2.03 (1.03–3.99). *P* = 0.039
**Study**	**Year**	**Cases**	**Stage**		**Assessment and treatment**	**Threshold**	**Outcomes**
**Muscle Invasive Bladder Cancer**							
Gondo et al. (47)	2012	189	cTa-4		Pre-RC	2.5	DSS, HR 1.95 (1.03–3.67). *P* = 0.038
Viers et al. (48)	2014	889	pT0-4/Nx-3		Pre-RC	2.7	RFS, HR 1.04 (1.01–1.08). *P* = 0.02 CSS, HR 1.04 (1.01–1.08). *P* = 0.01 OS, HR 1.03 (1.01–1.06), *P* = 0.01
Krane et al. (49)	2013	68	Recurrent T1HG and MIBC		Pre-RC	2.5	OS, HR 2.49 (1.14–6.09),
Hermanns et al. (50)	2014	424	pT0-4Nx/+M0		Pre-RC	3	5 y-RFS: 64 vs. 53%, *P* = 0.013 5 y-CSS: 75 vs. 57%, *P* < 0.001 5 y-OS: 64 vs. 43%, *P* < 0.001 RFS, HR 1.49 (1.12–2.00), *P* = 0.007 CSS, HR 1.88 (1.39–2.54), *P* < 0.001 OS, HR 1.67 (1.17–2.39), *P* = 0.005
Kang et al. (51)	2015	385	pT0-4N0-3M0		Pre-RC	2	CSS, HR 0.81 (0.38–1.7) OS, HR 0.97 (0.51–1.84)
Bhindi et al. (52)	2016	418	pT0-4Nx/+M0		Pre-RC	2.9	RFS, HR 1.52 (1.17–1.98), *P* = 0.002 CSS, HR 1.47 (1.20–1.80), *P* < 0.001 OS, HR 1.56 (1.16–2.10), *P* = 0.004
Kawahara et al. (53)	2016	74	pT0-4Nx/+M0		Pre-RC	2.38	OS, HR 4.62 (1.16–18.34), *P* = 0.030
Hirasawa et al. (54)	2016	136	cT1-4		Pre-RC	Continuous	CSS, HR 1.3 (1.1–1.5), *P* = 0.005
D'Andrea et al. (41)	2017	4435	pT0-4Nx/+M0		Pre-RC	2.7	RFS, HR 1.2 (1.1–1.3), *P* < 0.001 CSS, HR 1.2 (1.1–1.4), *P* < 0.001 OS, HR 1.1 (1.0–1.2), *P* = 0.01
Tan et al. (55)	2017	84	pT0-4Nx/+M0		Pre-RC	2.7	5 yr-DFS: 58 vs. 22%, *P* = 0.017 5 yr-OS: 60 vs. 23%, *P* = 0.008 RFS, HR 7.00 (1.71–28.60), *P* = 0.007
Kang et al. (56)	2017	385	pT0-4N0-3M0		Pre-RC	2.5	1 yr-OS, *P* = 0.018, 1 yr-CSS *P* = 0.001
Morizawa et al. (57)	2016	110	pT0-4Nx/+M0		Pre/Post-RC	2.6	RFS, HR 2.6 (1.1–6.0), *P* = 0.02 CSS, HR 2.6 (1.2–5.6), *P*= 0.01 OS, HR 2.8 (1.4–5.4), *P* < 0.01
Yoshida et al. (24)	2016	323	pT0-4N0/+M0		Pre/Post-RC	2.7/Kinetics	OS *P* < 0.001, (High < Low), OS *P* < 0.001, (HH < LL+HK+LH) OS, HR 2.56 (1.75–3.73) *P* < 0.001 (NLR change)
Buisan et al. (58)	2016	205	pT0-4Nx-2M0		Pre-RC/with NAC	2.26/ Continuous	PFS, HR 1.25 (1.10–1.42), *P* < 0.001 CSS, HR 1.27 (1.11–1.44), *P* < 0.001 OS, HR 1.12 (1.01–1.23), *P* < 0.021
Kaiser et al. (59)	2018	296	cT2-4aN0M0		Pre-NAC/ mid-NAC	3	Median DSS, 12.6 vs. 34.8 m, *P* = 0.002 Median OS, 19.4 vs. 44.0 m, *P* = 0.001
Ohtake et al. (60)	2016	23	M+		Pre-Chemotherapy/ Gemcitabine + Nedaplatin	4.14	PFS, *P* = 0.011 and OS, *P* = 0.045, (High < Low)
**Upper Urinary Tract Urothelial Cancer**							
Azuma et al. (61)	2013	137	pTa-4N0M0		Pre-RNU	2.5	5 yr-RFS, 74.3 vs. 30.45%, *P* < 0.001 5 yr-CSS, 81.3 vs. 29.4%, *P* < 0.001 RFS, HR 2.11 (1.02–4.46), *P* = 0.045 CSS, HR 3.06 (1.44–6.83), *P* = 0.003
Tanaka et al. (62)	2014	665	pTa-4Nx/+M0		Pre-RNU	3	5 yr-RFS, 69.2 vs. 57.0%, *P* < 0.001 5 yr-CSS, 77.3 vs. 60.2%, *P* < 0.001 RFS, HR 1.38 (1.02–1.87), *P* = 0.037 CSS, HR 1.47 (1.03–2.11), *P* = 0.036
Luo et al. (63)	2014	234	pTa-4N0M0		Pre-RNU	3	RFS, HR 2.47 (1.16–5.29), *P* = 0.020 CSS, HR 6.38 (1.75–23.31), *P* = 0.006
Dalpiaz et al. (64)	2014	202	pTa-4N0M0		Pre-RNU or segmental ureterectomy	2.7	CSS, HR 2.72 (1.25–5.93), *P* = 0.012 OS, HR 2.48 (1.31–4.70), *P* = 0.005
Altan et al. (65)	2017	113	pTa-4N0M0		Pre-RNU	2.9	5 yr-DFS, 83.2 vs. 30%, *P* < 0.001 5 yr-PFS, 53.7 vs. 18.3%. *P* < 0.001 DFS, HR 1.84 (1.10–3.09), *P* = 0.021 PFS, HR 2.90 (1.35–6.22), *P* = 0.006
Kishimoto et al. (66)	2017	100	pTa-4Nx/+M0		Pre-RNU	3.8	Intravesical-RFS, HR 2.49 (1.20–5.20), *P* = 0.015
Tan et al. (67)	2018	717	pTa-4Nx/+M0		Pre-RNU	2.5	RFS, HR 1.70 (1.31–2.20), *P* < 0.001 MFS, HR 1.67 (1.22–2.31), *P* = 0.002 CSS, HR 1.95 (1.42–2.69), *P* < 0.001 OS, HR 1.88 (1.42–2.50), *P* < 0.001

*BCG, Bacillus Calmette-Guérin; CSS, cancer specific survival; DFS, disease free survival; DSS, disease specific survival; HG, high grade; HR, hazard ratio; LG, low grade; MFS, metastatic free survival; MIBC, muscle invasive bladder cancer; OS, overall survival; PFS, progression free survival; PUNLMP, papillary urothelial neoplasm of low malignant potential; RC, radical cystectomy; RFS, recurrence free survival; RNU, radical nephroureterectomy; TURBT, transurethral resection of bladder tumor*.

Several studies have reported on the prognostic role of NLR in MIBC and metastatic BC (Table 2) (24, 41, 47–60). Furthermore, several studies have reported on the role of prognostic factors in survival outcomes of MIBC and metastatic UC (24, 41, 47, 48, 50, 52–56, 59, 60), while other studies have suggested that the postoperative stages or disease severity can be predicted (Table 3) (41, 48, 49, 58, 69–73). The majority of the studies reported a positive role of a high NLR on the prediction of worse OS and CSS rates. Furthermore, elevated NLR has been reported as an independent predictor of recurrence in several studies (32, 41, 50, 52, 55), with some reporting higher NLR values in patients with MIBC compared to those with NMIBC (69, 70). Other studies have reported on considering the NLR in predicting disease severity, such as extravesical involvement, upstaging, or LN involvement (48, 49, 55). Furthermore, some studies have considered the NLR to predict the pathologic complete response after chemotherapy or surgery (58, 72, 73). Seah et al. analyzed NLR kinetics before, during, and after NAC and reported differences in the patterns between the CR and non-responder groups (72). Although the role of the preoperative evaluation of NLR has been commonly discussed, some studies have assessed the prognostic value of changes in the ratio through repeated measurements over time (56). Kang et al. reported that postoperative NLR increases were associated with adverse pathological outcomes and were predictors of worse OS and CSS (51). Patients with consistently elevated NLR before and after surgery had worse OS and CSS compared to other patients (51). Yoshida et al. predicted patient prognosis by comparing the NLR before and after surgery (24). Patients with high and low NLRs were categorized based on their preoperative and postoperative values, respectively. Patients were divided into four groups according to the change in NLR before and after surgery. Patients with consistently high NLR before and after surgery demonstrated worse prognosis compared to other patients (24). The degree of change in the NLR before and after surgery was reported to be related to OS (24). In addition, Kiser et al. measured and analyzed the NLR at the beginning of and intermediately during NAC and reported differences in median DSS (12.6 vs. 34.8 m, *P* = 0.002) and median OS (19.4 vs. 44.0 m, *P* = 0.001) (59). One study analyzed the differences in the NLR over a period of 1–5 years after surgery and reported significant results of 1-year OS (*P* = 0.018) and 1-year CSS (*P* = 0.001) rates; however, there was no difference in the rates due to differences in the NLR from the 2nd year onwards (56). The majority of the studies have reported that the NLR before and after surgery or chemotherapy can be used to predict survival outcomes, pathological findings, and disease progression. Thus, measurement of the NLR before and after treatment is a reliable tool for predicting postoperative survival outcomes in patients with MIBC.

**Table 3:** The studies evaluating the role of neutrophil to lymphocyte of urothelial cancer pathologic staging.

**Study**	**Year**	**Cases**	**Stage**	**Assessment and treatment**	**Threshold**	**Predictable variable**	**Outcomes**
Can et al. (69)	2012	182	pT0-4N0M0	Pre-TURBT	2.57	MIBC possibility	OR 2.78 (1.38–5.59), *P* = 0.004
Lee et al. (70)	2015	226	pT0-4N0M0	Pre-TURBT	3.89	MIBC possibility	OR 8.24 (2.49–27.32), *P* = 0.001
Krane et al. (49)	2013	68	Recurrent T1 + MIBC	Pre-RC	2.5	Extravesical disease	OR 3.18 (1.09–9.79)
Potretzke et al. (71)	2014	102	pT0-4N0M0	Pre-RC	Continuous	≥pT3 upstaging Extravesical disease	OR 1.36 (1.01–1.84), *P* = 0.040 OR 1.5 (1.07–2.10), *P* = 0.020
Viers et al. (48)	2014	889	pT0-4N0M0	Pre-RC	2.7	Extravesical disease LN involvement	OR 1.07 (1.01–1.15), *P* = 0.030 OR 1.09 (1.02–1.16), *P* = 0.020
Buisan et al. (58)	2016	205	pT0-4Nx-2M0	Pre-RC	Continuous	pCR	OR 0.80 (0.64–0.99), *P* = 0.04
D'Andrea et al. (41)	2017	4435	pT0-4N0M0	Pre-RC	2.7	LN involvement	OR 1.9 (1.7–2.3), *P* < 0.001
Seah et al. (72)	2015	26	pT0-4N0-3	Pre-NAC/Mid/Pre-RC	Kinetics	NLR pattern during NAC	Different between pCR and non-responder, *P* = 0.038
Leibowitz-Amit et al. (73)	2016	55	pT2-4N0M0	Pre-NAC	Continuous	pCR	OR 0.48 (0.23–0.98), *P* = 0.05

*LN, lymph node; NLR, neutrophil to lymphocyte ratio; MIBC, muscle invasive bladder cancer; OR, odd ratio; OS, overall survival; pCR, pathologic complete response; RC, radical cystectomy; TURBT, transurethral resection of bladder tumor*.

Several studies have reported that preoperative assessment of the NLR can be considered to predict the survival outcomes in patients with UTUC (Table 2) (61–67), with a cutoff value between 2.5 and 3. Kishimoto et al. evaluated the predictive ability of the NLR before RNU on intra-bladder recurrence (66). The study reported that a high preoperative NLR was associated with an increased risk of recurrence of BC (HR 2.49, *P* = 0.015). The majority of the studies have reported a link between the CSS and RFS (101, 103, 105, 106). The European Association of Urology proposes the NLR as a prognostic factor for CSS in patients with UTUC (4). However, the evidence supporting prognostic role of NLR in UTUC remains low, and currently there are no guidelines of risk stratification (4).

Studies that evaluated the prognostic value of the NLR before chemotherapy for advanced or metastatic disease, wherein surgery was inappropriate, mainly reported on patients with BC and UTUC (Table 4) (55, 74–77), with a cutoff value of 3. Interestingly, one study evaluated the prognostic value of NLR kinetics before and after chemotherapy. A high NLR before chemotherapy helped predict poor survival outcomes (76).

**Table 4:** The studies evaluating the role of neutrophil to lymphocyte in urothelial cancer on chemotherapy.

**Study**	**Year**	**Cases**	**Site**	**Stage**	**Assessment and treatment**	**Threshold**	**Outcomes**
Rossi et al. (74)	2015	292	Bladder + Ureter	Advanced or Metastatic	Pre/Follow up-Chemotherapy	3/Kinetics	PFS, 2.76 (1.92–3.96), *P* < 0.001 OS, 3.15 (2.13–4.66), *P* < 0.001
Taguchi et al. (75)	2015	185	Bladder + Ureter	Metastatic	Pre-Chemotherapy	3	CSS, 1.48 (1.01–2.17), *P* = 0.043 OS, 1.49 (1.02–2.18), *P* = 0.040
Auvray et al. (76)	2017	280	Bladder + Ureter	Metastatic	Pre-Chemotherapy	3.2	OS, 1.36 (1.23–1.51), *P* < 0.001 PFS, 1.18 (1.05–1.33), *P* =0.005
Su et al. (77)	2017	256	Bladder + Ureter	Metastatic	Pre-Chemotherapy	3	OS 1.60 (1.21–2.31), *P* = 0.001
Tan et al. (55)	2018	150	Bladder	Advanced or Metastatic (cT4bN0M0 or TxN1-3M0 or TxNxM1)	Pre-Chemotherapy	3	OS 5.06 (2.88–8.88), *P* < 0.001

*CSS, cancer specific survival; HR, hazard ratio; OS, overall survival; PFS, progression free survival; RC, radical cystectomy; RFS, recurrence free survival; RNU, radical nephroureterectomy*.

## Albumin and Albumin-Globulin Ratio

Several studies have reported on the association of hypoalbuminemia with poor prognosis in patients with UC (Table 5) (49, 68, 72, 78–91). The cutoff value of albumin varies from 2 to 4 mg/L, although it is normally around 3.5 mg/L. Many studies have reported on the role of preoperative evaluation of albumin levels on the prognosis of patients. Hwang et al. reported that presence of hypoalbuminemia before chemotherapy was associated with a worse DFS (HR 2.04, *P* = 0.023) in patients with metastatic BC (83). Laurent et al. reported an increase in the 1-year mortality rate after chemotherapy (HR 3.06, *P* < 0.001) in patients with hypoalbuminemia before the treatment (82).

**Table 5:** The studies evaluating the role of albumin and Glasgow prognostic score in urothelial cancer.

**Study**	**Year**	**Case**	**Site**	**Stage**	**Assessment and treatment**	**Threshold**	**Outcomes**
**Albumin**							
Caras et al. (78)	2017	1374/4200	Bladder	NA	Pre-RC/Pre-TURBT	3.5 g/dL	OM, HR 1.49, *P* = 0.006 (RC); HR 2.71, *P* < 0.001(TURBT) OS, HR 4.0, *P* < 0.001(TURBT)
Lambert et al. (79)	2012	187	Bladder	pT0-4pN0-3M0	Pre-RC	3.5 g/dL	OS, HR 1.76, *P* = 0.04
Krane et al. (49)	2013	68	Bladder	Recurrent T1HG and MIBC	Pre-RC	3.5 g/dL	OS, HR 4.96 (2.18–11.67)
Ku et al. (80)	2015	419	Bladder	pT0-4N0/+M0	Pre-RC	3.5 g/dL	DSS, HR 1.79 (10.1–3.19), *P* = 0.046 OS, HR 1.67 (10.1–2.77), *P* = 0.047
Djaladat et al. (81)	2014	1964	Bladder	pT0-4N0-3M0	Pre-RC	3.5 g/dL	RFS, HR 1.68 (1.16–2.43), *P* < 0.001 OS, HR 1.93 (1.43–2.63), *P* < 0.006
Laurent et al. (82)	2017	197	Bladder	pT2-4N0/+M0/+	Pre-Chemotherapy	3.5 g/dL	1yr-mortality, HR 3.06 (1.81–5.17), *P* < 0.001
Hwang et al. (83)	2012	67	Bladder	M1	Pre-Chemotherapy	3.5 g/dL	DFS, HR 2.04 (1.10–3.78), *P* = 0.023
Ku et al. (68)	2014	181	Ureter	pTa-4N0/+M0	Pre-RNU	3.5 g/dL	DSS, HR 2.97 (1.25–7.03), *P* = 0.013 OS, HR 2.48 (1.18–5.21), *P* = 0.016
Seah et al. (72)	2016	101	Ureter	pTa-4N0/+M0	Pre-RNU	4.0g/dL	RFS, HR 4.40 (2.04–9.30), *P* < 0.001 OS, HR 3.37 (1.43–7.92), *P* = 0.005
Huang (84)	2017	425	Ureter	pTa-4N0/+M0	Pre-RNU	2.0 g/dL	CSS, HR 1.85 (1.14–3.00), *P* = 0.013 OS, HR 1.73 (1.12–2.70), *P* = 0.015
**Albumin/Globulin Ratio (AGR)**							
Niwa et al. (85)	2018	364	Bladder	pTa-T1N0M0	Pre-TURBT	1.6	RFS, HR 0.53 (0.36–0.78), *P* < 0.010 (low)
Liu et al. (86)	2016	296	Bladder	pT0-4N0/+M0	Pre-RC	1.60	5 yr-RFS 87.0 vs. 48.0%, *P* < 0.001 (high vs. low) Median CSS: 156.0 vs. 71.1 m *P* = 0.005 (high vs. low) RFS, HR 0.36 (0.17–0.75), *P* = 0.006 (low) CSS, HR 0.28 (0.11–0.68), *P* = 0.005
Liu et al. (87)	2018	189	Bladder	pT1-4N0/+M0	Pre-RC	1.55	PFS, HR 0.30 (0.15–0.61), *P* = 0.001 (low) CSS, HR 0.25 (0.10–0.58), *P* = 0.001 OS, HR 0.20 (0.09–0.47), *P* < 0.001
Zhang et al. (88)	2015	187	Ureter	pTa-4N0/+M0	Pre-RNU	1.45	OS, HR 0.45 (0.27–0.75), *P* = 0.002 (low) CSS, HR 0.47 (0.26–0.86), *P* = 0.015
Fukushima et al. (89)	2018	105	Ureter	pTa-4N0/+ M0	Pre-RNU	1.24	5 yr DFS, 90 vs. 60%, *P* < 0.001 (high vs. low) 5 yr OS, 89 vs. 65%, *P* < 0.001 DFS, 0.34 (0.10–0.95), *P* = 0.038 (low) OS, 0.24 (0.07–0.67), *P* = 0.006
Xu et al. (90)	2018	620	Ureter	pTa-4N0/+M0	Pre-RNU	1.45	5 yr-RFS, 58.4 vs. 38.3%, *P* < 0.001 (low vs. high) 5 yr-CSS, 72.8 vs. 48.9%, *P* < 0.001 5 yr-OS, 67.0 vs. 41.3%, *P* < 0.001 RFS, HR 1.32 (1.028–1.697), *P* = 0.029 (high) CSS, HR 1.50 (1.11–2.04), *P* = 0.010 OS, HR 1.40 (1.07–1.84), *P* = 0.015
Otsuka et al. (91)	2018	124	Ureter	pTa-4 N0/+M0	Pre-RNU	1.40	RFS, HR 3.96 (1.65–10.11), *P* = 0.002 (high) CSS, HR 5.69 (2.13–17.22), *P* < 0.001 OS, HR 3.12 (1.47–6.28), *P* = 0.003
**Glasgow Prognostic Score/Modified Glasgow Prognostic Score**							
Hwang et al. (83)	2012	67	Bladder	M1	Pre-Chemotherapy	GPS	OS, HR 7.00 (2.53–19.36), *P* = 0.001 (GPS2)
Qayyum et al. (92)	2013	68	Bladder	pTa-4N0M0	NA	mGPS	CSS, HR 1.78 (1.09–2.90), *P* = 0.020
Cho et al. (93)	2014	147	Ureter	NA	Pre-RNU	GPS	RFS, HR 6.86 (3.69–12.7), *P* = 0.001 (GPS1); HR 5.96 (3.10–11.4), *P* = 0.001 (GPS2)
Ferro et al. (94)	2015	1037	Bladder	pTa-4N0/+M0	Pre-RC	mGPS	5 yr-RFS 36 vs. 18 vs. 5%, *P* < 0.001 RFS, HR 1.54 (1.31–1.81), *P* < 0.001 (GPS1 vs. 2); HR 2.38 (1.86–3.05), *P* < 0.001 (GPS2)
Lucca et al. (95)	2016	310	Bladder	cTa-2N0M0	Pre-RC	GPS	NOC-UCB, HR 2.78 (1.52–5.09), *P* = 0.001 (GPS1); HR 5.37 (1.59–20.85), *P* = 0.008 (GPS2)
Wuethrich et al. (96)	2016	224	Bladder	pT0-4N0-3M0	Pre-RC	GPS	90-days mortality, HR 3.79 (1.29–11.14), *P* = 0.016 (GPS2)
Miyake et al. (97)	2017	117	Bladder	pT0-4N0/+M0	Pre-RC	mGPS	OS, HR 2.9 (1.5–5.8) *P*-0.002 (GPS1-2)
Inamoto et al. (98)	2017	574	Ureter	pT0-4N0M0	Pre-RNU	GPS	10 yr-CSS, 99.5 vs. 75.9%, *P* < 0.001 (GPS0 vs. 2) 10 yr-OS, 93.8 vs. 81.8%, *P* < 0.006 (GPS0 vs. 1); 93.8 vs. 67.6%, *P* = 0.001 (GPS0 vs. 2) OS, HR 1.54 (1.24–1.91), *P* < 0.01
Kimura et al. (99)	2019	1096	Bladder	pTa-1 N0M0	Pre-TURBT	mGPS	PFS, HR 2.06 (1.37–3.12), *P* = 0.001 (GPS1); HR 3.31 (1.40–7.87), *P* = 0.007 (GPS2)
Suyama et al. (100)	2019	74	Ureter	NA	Pre-RNU	GPS	OS, HR 2.28 (1.33–3.91), *P* = 0.003

*CSS, cancer specific survival; DFS, disease free survival; DSS, disease specific survival; GPS, Glasgow prognostic score; HR, hazard ratio; mGPS, modified Glasgow prognostic score; NOC-UCB, nomogram-confined urothelial carcinoma of the bladder; OM, overall morbidity; OS, overall survival; PFS, progression free survival; RC, radical cystectomy; RFS, recurrence free survival; RNU, radical nephroureterectomy; TURBT, transurethral resection of bladder tumor*.

AGR is the ratio of albumin and total proteins to albumin. The assessment of both albumin and globulin is believed to provide a greater prognostic insight than albumin alone. However, considering the limited number of studies, the utility of this combination remains to be established. Recent studies have reported on the prognostic effect of AGR in patients with UC (85–91).

## Glasgow Prognostic Score and Modified Glasgow Prognostic Score

The GPS and mGPS are a combination of CRP and albumin scores, representing methods designed to predict the prognosis of cancer patients. The GPS system is an indicator of the nutritional status of an individual based on systemic inflammation. McMillan et al. first introduced GPS for predicting the prognosis of patients with non-small cell lung cancer (101). Since then, GPS has proved to be useful in predicting the prognosis of patients with a variety of cancers, including colorectal, esophageal, lung, gastric, pancreatic, and liver cancers (101). Recently, few studies recorded the aforementioned scores before chemotherapy and surgery in patients with UC (Table 5) (83, 92–100). The majority of the preoperative evaluation studies were performed on patients undergoing RC and RNU. Preoperative GPS or mGPS were reported as factors predicting survival outcomes such as OS, CSS, and RFS. Ferro et al. conducted a study on 1,037 patients undergoing RC and reported longer RFS in the group with higher preoperative mGPS. Furthermore, the group with the highest mGPS demonstrated ~30% longer RFS compared to those with the lowest scores (94). Inamoto et al. conducted a study on 574 patients undergoing RNU and reported that patients with high GPS before surgery demonstrated 23.6% longer 10-years CSS and 12% longer 10-years OS compared to patients with low GPS (98). One study recorded preoperative GPS in patients undergoing TURB. Kimura et al. conducted a study on 1,096 patients undergoing TURB and reported that preoperative mGPS helped in the prediction of PFS after surgery (99). Another study recorded GPS before chemotherapy in patients with M1 BC. Hwang et al. reported that high GPS levels before chemotherapy were indicative of poor survival (HR 7.0, *P* = 0.001) (83).

## Platelet-to-Lymphocyte Ratio

Several studies have reported on the prognostic value of the preoperative assessment of the PLR (Table 6) (12, 42, 52, 64, 65, 70, 102, 103), wherein a high ratio has been reported to be associated with poor OS, CSS, and RFS. The cutoff value for PLR varies from 123 to 218. Interestingly, Kim and Ku categorized the PLR into three sections with two values, 150 and 300, but did not observe any relationship with the OS of the patients (12).

**Table 6:** The studies evaluating the role of platelet to lymphocyte in urothelial cancer.

**Study**	**Year**	**Cases**	**Site**	**Stage**	**Assessment and treatment**	**Threshold**	**Outcomes**
Kang et al. (42)	2017	1551	Bladder	pTa-1N0M0	Pre-TURBT	124	OS, CSS: Not significant
Lee et al. (52)	2015	226	Bladder	pTa-1N0M0	Pre-TURBT	218	OS: Not significant
Bhindi et al. (70)	2016	418	Bladder	pT0-4Nx/+M0	Pre-RC	150	RFS, CSS, OS: Not significant
Schulz et al. (102)	2017	665	Bladder	pT0-4N0/+M0	Pre-RC	28	CSS, HR, 1.4 (1.1–1.9), *P* = 0.022 OS, HR, 1.4 (1.0–1.8), *P* = 0.025
Son et al. (103)	2018	1137	Ureter	pTa-4N0/+M0	Pre-RNU	150	RFS, HR 1.32 (1.08–1.62), *P* = 0.007 CSS, HR 1.87 (1.21–2.92), *P* = 0.005
Kim and Ku (12)	2015	277	Ureter	pTa-4N0M0	Pre-RNU	<150, 150–300, >300	RFS, DFS: Not significant
Dalpiaz et al. (64)	2017	180	Ureter	pTa-4N0M0	Pre-RNU	150	CSS, HR 2.03 (1.04–3.93), *P* = 0.037 OS, HR 1.78 (1.04–3.05), *P* = 0.035
Altan et al. (65)	2017	113	Ureter	pTa-4N0M0	Pre-RNU	150	PFS, DFS: Not significant

*CSS, cancer specific survival; HR, hazard ratio; OS, overall survival; PFS, progression free survival; RC, radical cystectomy; RFS, recurrence free survival; RNU, radical nephroureterectomy*.

## Discussion

Since hematologic tests in cancer patients are basic and frequently repeated, SIR biomarkers using them can be easily obtained in cancer patients and used as economic and objective parameters. In the UC, in the system for predicting the existing prognosis or in nomogram and risk stratification studies, hematologic factors predicting pathological prognoses such as the number of tumors, tumor diameter, tumor grade, T stage, CIS, and LIV rather than biomarkers I mainly used them (12, 17, 26, 47, 95). However, in the case of clinical staging, the upstaging rate in pathological results after RC reaches 50%, and its accuracy is still low (12). Therefore, there is a need for other biomarkers to predict the prognosis of patients before treatment and to stratify risk, and for this purpose, SIR-related hematologic biomarkers can be a potential and effective factor. In the UC, the SIR biomarker is known to play an essential role in the progression and various oncologic outcomes, even though the inflammatory process develops cancer. In particular, CRP and NLR seem to be particularly useful for these oncologic outcomes and as predictors. And Albumin, AGP GPS, mGPS, PLR, etc. are potential factors, but they still lack data. The standard treatment for NMIBC is TURBT (2). However, due to frequent relapses and progression to MIBC after TURBT, regular examinations, and long-term follow-up are required (2). This repeated and long-term follow-up is putting a high economic burden on the patient (2). In addition, cystoscopy, the most representative test method, is an invasive test that causes significant pain and discomfort to the patient. Based on previous study results, patients with high CRP before surgery in NMIBC have a significant correlation with adverse outcomes in treatment outcomes and survival (18, 19). NLR had a high rate of pathologic upstaging in patients with increased NLR before TURBT (37–45), and the risk of progression to MIBC was also high (69, 70). SIR biomarkers can also be of great help in predicting the prognosis of NMIBC patients and determining the duration or method of follow-up and re-TURB. For MIBC, the standard treatment is RC. There are also various supplementary treatments such as chemotherapy, RT, and immunotherapy. In MIBC (3), patients with high CRP before surgery have a significant correlation with adverse outcomes in treatment outcomes and survival. And NLR was relatively low in complete response and pathologic T0 in patients with high NLR before RC or before NAC (47–60). And increased NLR levels during 1–3 months post RC were associated with adverse survival outcomes (12). The SIR biomarker may be helpful in MIBC patients considering the sequence and timing of treatment such as immediate RC and NAC after prognosis. And the patterns of change in NLRs before and after surgery can also provide useful information for determining additional target groups, such as adjuvant chemotherapy, radiation therapy, and immunotherapy.

In addition to the systemic inflammatory response, CRP is a biomarker that predicts the outcome of IL-2 or IFN-α immunotherapy and has been considered in the evaluation of patients with renal cancer (104). Patients with normal CRP have been reported to demonstrate good tolerance and adherence to immunotherapy (104). The scope of immunotherapy is expanding in patients with UC, and CRP could be considered a factor in predicting the prognosis and compliance to treatment.

The limitations of SIR biomarker's research at UC are: First, there is no objective and clear cut off value. Second, unlike pathological prognostic factors, SIR biomarkers may be affected by other inflammatory diseases or physical conditions other than the patient's cancer. And third, due to the nature of the research, most studies are retrospective and lack of large-scale prospective research results.

## Conclusion

In this review, we discussed the applicability of CRP, AGR, albumin, GPS, mGPS, NLR, and PLR as SIRs in patients with UC. These SIRs have proven to be useful during preoperative evaluations in the treatment and risk assessment of patients with UC. However, there is a need to develop uniform classification criteria with a consensus regarding the use of these SIRs in clinical settings. We believe that large prospective studies are needed on this subject based on observations of this retrospective review.

## Author Contributions

HY and JK contributed to the concept, project planning, and contributed significantly to the writing of the manuscript. JK contributed significantly in the editing of the final manuscript. All authors contributed to the article and approved the submitted version.

## Conflict of Interest

The authors declare that the research was conducted in the absence of any commercial or financial relationships that could be construed as a potential conflict of interest.
